# Genetic Determinants of Colonic Diverticulosis—A Systematic Review

**DOI:** 10.3390/genes16050581

**Published:** 2025-05-15

**Authors:** Piotr Nehring, Adam Przybyłkowski

**Affiliations:** Department of Gastroenterology and Internal Medicine, Medical University of Warsaw, Banacha 1a, 02-097 Warsaw, Poland; piotr.nehring@wum.edu.pl

**Keywords:** colon, colonic diverticulosis, genetic associations, genetic predisposition, genetic variant

## Abstract

**Background**: Colonic diverticulosis is a common condition, particularly in the elderly population. While dietary habits, obesity, smoking, and physical inactivity contribute to its pathogenesis, emerging evidence highlights a genetic predisposition affecting extracellular matrix (ECM) remodeling, inflammation, and connective tissue integrity. The aim of this systematic review was to summarize genetic determinants of colonic diverticulosis. **Methods**: The PubMed^®^ database was searched for original studies in humans. The inclusion criteria were named genetic factor and confirmed diverticulosis. Patients with diverticulitis and diverticular diseases were excluded from this review. **Results**: Out of 137 publications, 10 articles met the inclusion criteria: six large association studies (GWAS) and four cross-sectional studies. The genes regulating ECM turnover, including *TIMP1*, *MMP3*, and *MMP9*, are involved in diverticulosis development. The *TIMP1* (*rs4898*) T allele has been associated with increased susceptibility, potentially due to its role in ECM remodeling. Similarly, *MMP3* (*rs3025058*) and *MMP9* (*rs3918242*) polymorphisms contribute to altered collagen degradation. The *COL3A1* (*rs3134646*) variant coding modified collagen type III may promote diverticular formation. Other genes, such as *ARHGAP15* (*rs4662344*, *rs6736741*), affect cytoskeletal dynamics. Identified in GWAS studies, gene candidates may be grouped into blood group and immune system-related genes (*ABO*, *HLA-DQA1*, *HLA-H*, *OAS1*, *TNFSF13*, *FADD*), extracellular matrix and connective tissue genes (*COL6A1*, *COLQ*, *EFEMP1*, *ELN*, *HAS2*, *TIMP2*), signaling and cell communication (*BMPR1B*, *WNT4*, *RHOU*, *PHGR1*, *PCSK5*), nervous system and neurodevelopment (*BDNF*, *CACNB2*, *GPR158*, *SIRT1*, *SCAPER*, *TRPS1*), metabolism and transporters (*SLC25A28*, *SLC35F3*, *RBKS*, *PPP1R14A*, *PPP1R16B*), lipids and cholesterol (*LDAH*, *LYPLAL1*, *STARD13*), transcription and gene regulation (*ZBTB4*, *UBTF*, *TNRC6B*), apoptosis (*FADD*, *PIAS1*), and poorly characterized genes (*C1TNF7*, *ENSG00000224849*, *ENSG00000251283*, *LINC01082*, *DISP2*, *SNX24*, *THEM4*, *UBL4B*, *UNC50*, *WDR70*, *SREK1IP1*). **Conclusions**: There are a number of gene variants that probably predispose to colonic diverticulosis. Detailed characterization of the multigene background of diverticulosis will enable appropriate therapeutic or preventive interventions in the future.

## 1. Introduction

The prevalence of colonic diverticulosis varies in populations around the globe. It is generally recognized that diverticulosis prevalence is higher in the elderly population. In Eastern populations, colonic diverticulosis prevalence was estimated to be 1.97%, and in 85.3% of cases, it was right-sided [1], whereas in Western populations, it is estimated to affect as much as 60% of patients over 60 years of age [2]. In a European study, colonic diverticula were found in 29.6% of adults during colonoscopy, with equal prevalence in both sexes [3]. While often asymptomatic, diverticulosis can progress to symptomatic diverticular disease or diverticulitis, causing significant morbidity and healthcare burden [1,2].

The pathogenesis of colonic diverticulosis is multifactorial, involving an interplay between increased colonic pressure, structural weakness of the colon wall, altered gut microbiota, inflammation, and age-related changes [4,5]. One of the primary factors contributing to the formation of diverticula is high intraluminal pressure. The colon’s muscular wall generates significant pressure during peristalsis to move the stool. In a low-fiber diet, containing usually red meat and processed food, stool tends to be harder and more difficult to pass, thus stimulating prolonged periods of increased pressure in the colon. This pressure can eventually force the mucosal and submucosal layers of the colon to herniate through weak points in the muscular layer, resulting in the formation of diverticula [4]. Recent research has highlighted the significance of dietary habits, stressing the protective effect of fiber-rich diets in preventing both the onset and progression of diverticular disease [6]. Beyond dietary factors, emerging evidence suggests a potential link between lifestyle and diverticulosis. Sedentary lifestyle and obesity have been recognized as independent risk factors for diverticular disease [7]. The mechanisms behind these associations are still under active investigation, with hypotheses spanning from the modulation of gut microbiota to systemic inflammation. The formation of diverticula is also influenced by structural weakness in the colonic wall. According to Hansen and Stock’s classification of diverticular disease, asymptomatic diverticulosis (stage 0) may progress to uncomplicated diverticulitis (stage I), and further to complicated diverticulitis (stage II) with presence, of phlegmon, abscess or sealed or free perforation, and finally to chronic recurrent diverticulitis (stage III) [8]. Similarly, stages of diverticulosis progression may be identified. The muscular layer of the colon, particularly the circular muscle fibers, may exhibit defects or thinning that predispose to herniation under high pressure. The wall of the colon is structurally weak at points, where blood vessels (vasa recta) penetrate the muscular layer to supply the blood (stage 1). These vessels create areas of inherent weakness, which can become the site of diverticulum formation under increased pressure (stage 2). The role of connective tissue in the pathogenesis of diverticulosis is crucial. Alterations in the production or breakdown of collagen and elastin fibers within the muscular layer of the colon can compromise the structural integrity and elasticity of the colonic wall, increasing its susceptibility to herniation under mechanical stress [9,10]. Some genetic factors may predispose individuals to these connective tissue alterations, leading to an increased risk of diverticular formation. Genetic factors involved in extracellular matrix (ECM) remodeling, inflammation, and connective tissue integrity are thought to modulate susceptibility. Several studies have shown that individuals with first-degree relatives affected by diverticulosis are at a higher risk, and certain gene polymorphisms have been linked to the condition [2,11].

The aim of this review was to systematize the available knowledge about genetic risk factors of colonic diverticulosis.

## 2. Methods and Findings

### 2.1. Criteria for Considering Studies for This Review

Exclusively original studies in humans were searched. The included studies were required to assess patients with diverticulosis and name the identified genetic factor (e.g., polymorphism, mutation, genetic variant). The present systematic review included all GWAS studies and studies with named SNPs. The studies on diverticulitis and diverticular disease were excluded. The genetic factors that contribute to the formation of diverticula may differ from those that lead to symptomatic uncomplicated diverticular disease or diverticulitis. Each of these conditions involves a distinct anatomical and pathological process, which is not clearly distinguished in the ICD-10 classification. Additionally, clinicians often use the terms “diverticulosis” and “diverticular disease” interchangeably. For this reason, such studies were excluded, as they conflate the risk factors for diverticula formation with those for diverticulitis—factors that are also shared by other infection-based diseases.

### 2.2. Search Strategy

Two independent reviewers (PN and AP) conducted a search in the PubMed^®^ database (National Library of Medicine, Bethesda, MD, USA) following a predefined research strategy. The following terms were searched in all fields in the query box: ‘diverticulosis’, ‘colon’, and ‘genetic’. Given the limited number of studies, no restrictions were applied regarding publication date or language. This review includes studies published and accessible up until the end of April 2025. There were no disagreements between the reviewers regarding study inclusion, and all reviewers unanimously approved the final selection of articles. The items marked as review articles by the PubMed^®^ database were automatically recognized as illegible for this review. Data were collected independently by all reviewers manually. The searched outcomes were named genetic variant, risk allele, number of participants, population (ethnicity), mean (or median) age, sex, effect measured with odds ratio (OR) (if available), method of genotyping, possible SNP’s in silico effect. This manuscript was prepared in accordance with the PRISMA guidelines [12].

### 2.3. Research Results

**Figure 1:**
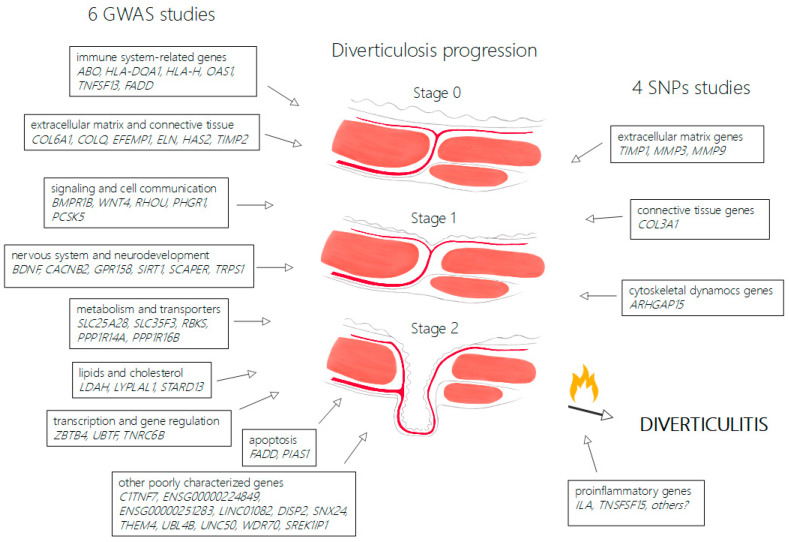
Overview of genetic determinants in colonic diverticulosis and proposed disease progression. Figure 1 demonstrates progression stages of diverticulosis from normal wall of large intestine (stage 0) to weakening of tissue in site of vasa recta passing muscular layer (stage 1) and full diverticula formation (stage 2). The left side of the figure groups genes identified in GWAS studies and the right side groups genes identified in SNP studies, according to possible mechanisms. Diverticulitis may complicate diverticulosis in carriers of predisposing proinflammatory genes.

In total, 10 studies meeting the inclusion criteria for this review were found: 6 large association studies based on GWAS search strategy and 4 cross-sectional studies. The PRISMA flowchart for this review is provided in Supplementary Figure S1. For better visualization, this review was divided into sections separately discussing genome-wide association search strategy studies’ results, the role of extracellular matrix, cytoskeletal dynamics genes and proinflammatory cytokines. Due to the heterogeneity of the studies, their construction, results and collected data, it was not possible to conduct a meta-analysis. The summary of the search results and progression stages of diverticula formation is shown Figure 1. The summary and comparison of selected studies on genetic predisposition to colonic diverticulosis is shown in Table 1.

#### 2.3.1. Genome-Wide Association Search Strategy

The whole-genome sequencing (WGS) method allows researchers to identify millions of sequence variants in the human genome. With the beginning of the post genome-wide association studies (GWAS) era, only *ARHGAP15*, *FAM155A*, and *COLQ* were linked with colonic diverticula and diverticulitis [13]. Maguire et al., in a large association study based on a genome-wide search strategy for possible loci associated with colonic diverticulosis, discovered another 42 possible genetic variants, 39 of which were newly identified [14]. Genes identified in Maguire et al.’s study are involved in immune function, extracellular matrix turnover, cell adhesion, membrane transport, and intestinal motility. A phenome-wide association study of these 42 variants suggests a shared genetic background for diverticular disease, obesity, and hernias. Schafmayer et al. discovered 12 novel loci connected with diverticular disease or diverticulitis indicating neuromuscular, connective tissue dysfunction, and epithelial disease mechanisms [15].

The recent transcriptomic study by Seo et al. on colonic specimens identified 38 genes with differing expression levels and 17 genes with altered transcript usage associated with diverticulosis, suggesting that tissue remodeling plays a key role in the formation of diverticula [16]. The formation of diverticula was primarily linked to stromal and epithelial cells in the colon, including endothelial cells, myofibroblasts, fibroblasts, goblet cells, tuft cells, enterocytes, neurons, and glial cells. Seo et al.’s study highlighted five genes *CCN3*, *CRISPLD2*, *ENTPD7*, *PHGR1*, and *TNFSF13* as having potential causal effects on diverticulosis. Notably, *ENTPD7* was found to be upregulated in individuals with diverticulosis [16]. Additionally, the grade of diverticulosis was associated with genetic susceptibility to diverticulitis. Seo et al.’s study suggested that tissue remodeling is a key process in the development of diverticula, and individuals with a higher genetic predisposition to diverticulitis tend to have a greater number of diverticula observed during colonoscopy [16].

Another GWAS-based study on a Korean population of patients with right-sided diverticulosis identified another nine new loci possibly correlated with colonic diverticula formation [17]. Choe et al.’s study pointed at genetic variants in wingless-type MMTV integration site family member 4 (*WNT4*) and Ras homolog family member U (*RHOU*), and (*OAS*) 1/3 genes [17,18]. *WNT4* was associated with vascular smooth muscle cell proliferation [19]. The RHOU mediates the WNT signaling pathway, which regulates cell morphology, cytoskeletal organization and cell proliferation [20]. The OAS family of proteins is induced by interferon and is associated with the antiviral and apoptotic responses [21]. Choe et al.’s study may indicate that right-sided diverticulosis may be associated with the regulation of vessels’ formation and inflammation [22].

#### 2.3.2. Role of Extracellular Matrix (ECM)

Connective tissue dysfunction contributes to remodeling the colonic mucosa and submucosa into more susceptible to herniation. Alterations in collagen composition notably impact the mechanical strength of connective tissue in individuals with diverticulosis and chronic inflammation [10]. Additionally, collagen fibrils in the left colon tend to decrease in size and become more densely packed as individuals age, especially within the diverticula of the colon [9].

##### Tissue Inhibitors of Metalloproteinases (TIMPs)

Tissue inhibitors of metalloproteinases (TIMPs) regulate the activity of matrix metalloproteinases (MMPs), which are involved in the degradation of ECM proteins. TIMP1 plays a crucial role in maintaining the balance of ECM turnover by inhibiting MMPs that degrade collagen and other structural components. Increased expression of TIMP1 may impair ECM remodeling, potentially contributing to the formation of weak spots in the colonic wall, predisposing individuals to diverticula formation.

TIMP1 expression is elevated in colon diverticula as well as in colorectal cancer [23]. Studies demonstrated that in the colonic mucosa and submucosa of patients with complicated diverticulosis, there is an increase in mRNA of TIMP1 and TIMP2 compared to healthy subjects [24]. Moreover, the levels of TIMP1 and TIMP2 mRNA are elevated in the colonic muscular layer in patients with diverticulosis, which suggests an association of TIMP expression alteration with diverticula formation [24]. The *TIMP1* variant 372 T/C (rs4898) is a single-nucleotide polymorphism (SNP) caused by the substitution of thymine with cytosine at position +372, leading to a silent mutation in exon 5 of the *TIMP1* gene. The C allele of *TIMP1* rs4898 is associated with arterial hypertension in Malaysian males [25], while the T allele is linked to higher serum TIMP1 levels and increased mortality in individuals with sepsis [26,27].

In male patients with diverticulosis, the allele T of *TIMP1* (rs4898) was more prevalent than in healthy controls [28]. The *TIMP1* rs4898 T allele has been associated with higher TIMP1 concentrations in serum [29], and this allele appears to be more prevalent in individuals with diverticulosis [29]. Additionally, *TIMP1* expression is elevated in the colonic mucosa of patients with diverticulitis, suggesting that TIMP1 might also play a role in the inflammatory process that complicates diverticulosis. TIMP1 is mostly responsible for inhibiting proMMP9 [30]. In vivo, MMP9 activity is critical in remodeling components of the ECM, including collagen IV and laminin in the basement membrane [31]. It was shown that TIMP1 serum concentration was elevated in individuals with rs4898 allele T in *TIMP1* [26]. An increase in the TIMP1 level may interplay with proMMP9 levels, which plays a crucial role in ECM breakdown and may predispose individuals with rs4898 allele T in *TIMP1* to diverticulosis. Moreover, TIMP1, MMP1 and MMP2 concentrations in colon were higher in tissue samples with diverticula compared to not-affected tissue [32]. This leads to the conclusion that rs4898 allele T *TIMP1* may predispose males to diverticulosis. Moreover, *TIMP2 rs1973232* was also identified in GWAS-based analysis to be linked with diverticular disease [15].

##### Matrix Metalloproteinases (MMPs)

Matrix metalloproteinases (MMPs) are a family of zinc-dependent enzymes that play a crucial role in the breakdown of ECM components. Among the MMP family, MMP3, MMP9, and MMP12 are particularly relevant to the pathogenesis of diverticulosis. These enzymes are responsible for the degradation of collagen, elastin, and other ECM proteins, which contribute to the structural integrity of the colonic wall [14].

MMP3, known as stromelysin, is produced by fibroblasts and myofibroblasts and is capable of degrading various ECM components, including type IV collagen, laminin, and proteoglycans. The *MMP3* (rs3025058) 5A/5A genotype was linked to the prevalence of diverticulosis [29]. The 6A allele of this polymorphism has been shown to have reduced transcriptional activity, which could affect the balance of ECM degradation in the colon [29].

MMP9, also known as neutrophil gelatinase, is involved in the breakdown of elastin and collagen type IV. It is produced by various cell types, including neutrophils and fibroblasts, and its activity is tightly regulated by TIMPs. The *MMP9* gene contains a polymorphism, rs3918242, which has been linked to diverticulosis. The presence of the T allele of this polymorphism may enhance the activity of MMP9, contributing to increased ECM remodeling in the colon [14]. The allele T of *MMP9* (rs3918242) was notably more common in individuals with diverticulosis compared to the healthy control group [29].

MMP12, another important member of the MMP family, is primarily produced by macrophages and is involved in the degradation of elastin. Although MMP12 plays a role in ECM remodeling, its involvement in diverticulosis is considered less significant compared to MMP3 and MMP9 [28,29].

##### Collagen and Connective Tissue Integrity (COL)

Collagen is a major component of the ECM, providing structural support to tissues. Type III collagen, encoded by the *COL3A1* gene, is particularly important in tissues subjected to mechanical stress, including the colon. This gene in chromosome 2 encodes for collagen type 3 α 1 chain. Variations in the *COL3A1* gene have been associated with an increased susceptibility to diverticulosis [33]. The *COL3A1* polymorphism, rs3134646, has been shown to be more frequent in patients with diverticulosis compared to healthy controls [33]. This variant may result in transcripts in regulatory regions possibly playing the role of enhancer. *COL3A1* (rs3134646) was also linked to gastroesophageal reflux disease, and hiatal hernia [33,34]. The authors’ own research confirmed that allele C of *COL3A1* (rs3134646) may be related to diverticulosis, as this variant was more frequent in individuals with colonic diverticulosis [35]. This genetic variant may influence collagen synthesis and crosslinking, affecting the colon’s mechanical properties and predisposing patients to diverticula formation [36]. In individuals affected with colonic diverticulosis, collagen synthesis is increased and collagen crosslinking is altered [36]. These changes contribute to the mechanical instability of the colonic wall, making it more prone to the formation of diverticula. Also important, collagen remodeling is regulated by MMPs and TIMPs, linking genetic factors involved in ECM turnover to the pathogenesis of diverticulosis [14,34].

#### 2.3.3. Cytoskeletal Dynamics Genes (ARHGAP15)

ARHGAP15, Rho GTPase activating protein 15, a protein encoded by the *ARHGAP15* gene in chromosome 2, plays a key role in regulating actin dynamics and cell movement, migration, and adhesion. Overexpression of *ARHGAP15* causes an increase in actin stress fibers and cell contraction. This gene contains a variant known as rs4662344, which has been linked to diverticulosis [13]. Moreover, another variant in this gene was identified in one of the GWAS-based studies, namely rs6736741, to be linked with diverticulosis [37]. It contributes to cell adhesion and cytoskeletal organization, both crucial for preserving the integrity of the colonic lining. Changes in *ARHGAP15* expression may influence how cells respond to mechanical stress, potentially leading to the formation of diverticula [38]. Research indicates that the T allele of this polymorphism is associated with a higher risk of diverticulosis, suggesting that *ARHGAP15* may impact susceptibility to colonic diverticula by affecting extracellular matrix remodeling and inflammation [13,35,39].

#### 2.3.4. Proinflammatory Cytokines (IL, TNF)

The *IL1A* gene, located on chromosome 2, encodes interleukin-1 α. The T allele of the *IL1A* variant rs1800587 (C-889T) has been linked to central obesity and metabolic syndrome in individuals with coronary heart disease, as well as an increased risk of intervertebral disc disease [40,41]. Since this allele is associated with obesity—a known risk factor for colonic diverticula formation—it was considered a candidate for analysis. However, in the authors’ own research, there were no significant differences in allele distribution for this variant, possibly due to the relatively low prevalence of obesity in the studied population [35].

The *TNFSF15* gene, located on chromosome 9, encodes tumor necrosis factor superfamily member 15, a key regulator of immune function and inflammation. It is involved in both normal immune responses and the development of inflammatory diseases. The *TNFSF15* variant rs7848647 has been linked to inflammatory bowel diseases, irritable bowel syndrome, and diverticulitis [42,43,44,45,46]. However, previous studies do not show its link to diverticulosis itself [35].

It seems that genes encoding proinflammatory cytokines are more likely to be linked to diverticulitis than diverticulosis.

**Table 1:** Summary and comparison of selected studies on genetic predisposition to colonic diverticulosis.

Study	Gene	Risk Allele	Population	Age	Ethnicity	Method	DICA Score	OR	SNP’s Effect *	Annotations
Nehring et al. (2025) [35]	*COL3A1 rs3134646* *ARHGAP15 rs4662344*	C T	134 cases; 189 controls	Mean 65.31 ± 13.31 vs. 62.80 ± 11.81	Poles	RT-PCR	1	1.90 3.44	Not found Not found	Both sexes
Seo et al. (2024) [16]	*JOSD1* *ENTPD7* *SREK1IP1* *TNSF13* *SIRT1* *AP3M1* *PHGR1* *CRISPLD2* *CCN3* *HLA-H* *LDAH* *THEM4*	N/A	172 cases; 232 controls	Mean 55.7 ± 7.30 vs. 53.5 ± 6.24	Self-reported: 80% white 20% black	Transcriptomics, GWAS, cis-eQTL	61% distal colon, 35% distal and proximal colon, 4% proximal colon		N/A	Both sexes
Nehring et al. (2023) [29]	*MMP9 rs3918242* *MMP3 rs3025058*	T 5A/5A	59 cases; 75 controls	Mean 64.5 ± 12.6 vs. 60.9 ± 12.6	Poles	RT-PCR	1	2.62 2.25	Not found Not found	Both sexes
Joo et al. (2023) [37]	*ARHGAP15 rs6736741*	C	12,577 cases; 9200 controls	Cases 62.5 vs. N/A	USA	GWAS, RT-qPCR	N/A	1.17	Not found	Both sexes
Nehring et al. (2021) [28]	*TIMP1 rs4898*	T	100 cases; 120 controls	Mean 64.3 ± 12.4 vs. 61.8 ± 11.1	Poles	RT-PCR	1	N/A	Tolerated	Males
Choe et al. (2019) [17]	*WNT4 rs11799918* *WNT4 rs75637000* *WNT4 rs2473253* *RHOU rs72751907* *RHOU rs4993975* *RHOU rs11583565* *RHOU rs11580020* *OAS1*, *OAS3 rs11066453* *OAS1*, *OAS3 rs2072134*	A T T T C T A G A	893 cases; 1075 controls Replication: 346 cases; 305 controls	61.5 ± 5.4 vs. 54.6 ± 8.9 ^#^	Koreans	GWAS; RT-PCR	Right-sided	1.39 1.366 1.415 0.657 0.676 0.676 0.672 0.694 0.676	Not found Not found Not found Not found Not found UTR_3 UTR_3 Not found UTR_3	
Schafmayer et al. (2019) [15] Listed only newly discovered comparing to Maguire et al. (2018) [14]	*SCAPER rs2056544* *CTAGE1 rs9960286* *TNRC6B rs6001870* *C1TNF7 rs4132788* *PIAS1 rs387505* *SNX24 rs34126945* *TIMP2 rs1973232* *PPP1R16B rs208814* *HLA-DQA1 rs7990* *PLEKHA1 rs139760870* *ITBP1 rs6714546* *STARD13 rs14473813*	G G C T T G G A A A A A	UK Biobank: 31,964 cases; 419,135 controls European replication: 3893 cases; 2829 controls	Median 72 (68–76) vs. 68 (60–73)	European Germans, Austrians, Lithuanians, Swedes	GWAS, RT-PCR	N/A	0.93 1.14 1.09 1.10 1.10 0.93 1.01 1.02 1.02 0.91 1.03 0.98	Not found Not found Not found Not found Not found Not found Not found Not found Tolerated Not found Not found Not found	Both sees
Reichert et al. (2018) [33]	*COL3A rs3134646*	A	422 cases; 285 controls	Median 68 (32–95) vs. 57 (19–83) ^#^	Germans, Lithuanians	RT-PCR	N/A	1.82	Not found	Males
Maguire et al. (2018) [14]	*ARHGAP15 rs6734367* *SLC35F3 rs4333882* *COLQ rs7609897* *GPR1581 rs7086249* *EFEMP1 rs1802575* *PPP1R14A rs11667256* *BDNF rs962369* *FAM185A*, *rs6949391* *LYPLAL1 rs61823192* *FAM155A rs9520344,* *rs11619840* *SLC25A28 rs7098322* *WDR70 rs10472291* *ABO rs582094* *COL6A1 rs75434097* *LINC01082 rs2280028* *P2RY12 rs9856118* *DISP2 rs71472433* *CRISPLD2 rs2131755* *ENSG00000224849 rs4839715* *UNC50 rs148376933* *NOV rs1381335* *S100A10 rs61814883* *UBTF rs8074740* *SHFM1 rs3113037* *CALCA rs12293535* *FADD rs875107,* *rs72945112* *ELN rs3823878* *CWC27 rs10471645* *CACNB2 rs1888693* *HAS2 rs4871180* *TRPS1 rs2049865* *BMPR1B rs1544387* *ENSG00000251283 rs11934833* *HLX rs2784255* *PCSK5 rs10120333* *ZBTB4 rs12942267* *NT5C1B rs62126581* *UBL4B rs115490395* *EDEM1 rs2470653* *RBKS rs10173528* *ISL2 rs2056544,* *rs10519134* *GTPBP1 rs138699*	G G T C C T C T T A A T A T A A G C G A T A A T A A T A C A T A T G C G T A A A T A A A	UK Biobank: 27,444 cases; 382,284 controls MGI: 2572 cases; 28,649 controls		European European ancestry in USA	GWAS, RT-PCR	N/A	N/A	Not found Not found Not found Not found UTR_3 Not found Not found Not found Not found Not found Not found Not found Not found Not found Not found Not found Not found Not found Not found Not found Not found Not found Not found Not found Not found Not found Not found Not found Not found Not found Not found Not found Not found Not found Not found Not found Not found Not found Not found Not found Not found Not found Not found Not found Not found	Both sexes
Sigurdsson et al. (2017) [13]	*COLQ* rs7609897 *ARHGAP15* rs4662344	T T	8734 cases; 248,971 controls	N/A	Icelanders Danes	GWAS, RT-PCR	N/A	0.87 1.23	Not found Not found	Both sexes

* SNP’s effect according to PolyPhen2 and SIFT database; MGI—Michigan Genomics Initiative; N/A—not available; UTR—untranslated region; ^#^—marks significant differences (*p* < 0.05).

## 3. Discussion

Colonic diverticulosis is a complex disease, with both environmental and genetic factors contributing to its pathogenesis. The role of genetic factors remodeling ECM, such as *TIMP*, *MMP*, *COL*, and *ARHGAP*, highlights the importance of collagen metabolism in the development of diverticulosis. Genetic polymorphisms in these genes may alter the balance of ECM degradation and synthesis, leading to structural changes in the colonic wall that predispose individuals to diverticula formation.

According to the results of GWAS, several candidates are identified, including blood group and immune system-related genes (*ABO*, *HLA-DQA1*, *HLA-H*, *OAS1*, *TNFSF13*, *FADD*), extracellular matrix and connective tissue genes (*COL6A1*, *COLQ*, *EFEMP1*, *ELN*, *HAS2*, *TIMP2*), signaling and cell communication (*BMPR1B*, *WNT4*, *RHOU*, *PHGR1*, *PCSK5*), nervous system and neurodevelopment (*BDNF*, *CACNB2*, *GPR158*, *SIRT1*, *SCAPER*, *TRPS1*), metabolism and transporters (*SLC25A28*, *SLC35F3*, *RBKS*, *PPP1R14A*, *PPP1R16B*), lipids and cholesterol (*LDAH*, *LYPLAL1*, *STARD13*), transcription and gene regulation (*ZBTB4*, *UBTF*, *TNRC6B*), apoptosis (*FADD*, *PIAS1*), and poorly characterized genes (*C1TNF7*, *ENSG00000224849*, *ENSG00000251283*, *LINC01082*, *DISP2*, *SNX24*, *THEM4*, *UBL4B*, *UNC50*, *WDR70*, *SREK1IP1*). This indicates that diverticulosis is a multifactorial disease, like alopecia, obesity, type 2 diabetes, hypercholesterolemia, and meets the criteria of a civilization disease. However, GWAS-based results are subject to certain errors of underestimation or overestimation of the population, which stem from the system of reporting diseases based on the ICD-10 and ICD-9 codes. In the worst-case scenario, data may come not from healthcare system reports but from medical interviews or patient questionnaires. It is often unclear whether a colonoscopy was performed or if only a CT scan was used. Most importantly, there is no standardized assessment of diverticulosis grading and location using available scales such as the DICA score.

Therefore, the GWAS results may be regarded as preliminary and should be confirmed in case–control or cross-sectional studies. In cross-sectional studies, only evidence of the relationship between variants of genes responsible for intestinal wall structure was found, which is consistent and expected, while the GWAS results suggest the influence of coding genes not directly related to the structure and function of the colon. Crucially, the genetics predisposing to diverticula formation may not be the same as the genetics predisposing to symptomatic uncomplicated diverticular disease or diverticulitis. Each of these conditions represents a distinct anatomopathological issue, which is not clearly differentiated in the ICD-10 and ICD-9 classifications. Furthermore, the terms “diverticulosis” and “diverticular disease” are often used interchangeably by clinicians. And for this reason, it was intended to exclude such studies because they may confound risk factors for the development of diverticula with risk factors for diverticulitis, which are shared with other infectious diseases. Asymptomatic diverticulosis represents the purest anatomical form, whereas diverticular disease can mimic and overlap with IBS symptoms, raising questions about cause-and-effect relationships. Meanwhile, diverticulitis, diagnosed based solely on abdominal CT, may be confused with complications of other conditions. Endoscopic verification and assessment using available scoring systems appear essential for a reliable diagnosis. The search for the genetic causes of diverticulitis and diverticular disease should be conducted separately from investigations into the causes of diverticula formation as anatomical changes in the colonic wall.

The search being limited to one database may be regarded as a limitation of this systematic review. Due to the heterogeneity of the studies included in this review, it lacks a mathematical analysis and synthesis of the results. The authors recognize that the limited number of studies included may result in an interpretation focused on the available results and a lack of a broader perspective.

Well-designed studies will be able to uncover the genetic determinants of diverticulosis and to explore potential therapeutic targets based on these findings.

## Data Availability

No novel datasets or data repositories were generated or disseminated in the course of conducting this systematic review beyond the data explicitly reported within the manuscript.

## Supplementary Materials

The following supporting information can be downloaded at: https://www.mdpi.com/article/10.3390/genes16050581/s1, Figure S1: PRISMA chart for Genetic Determinants of Colonic Diverticulosis.
