# Supplementary material for: Genetic Determinants of Colonic Diverticulosis—A Systematic Review
Source: Genes (Basel). 2025 May 15;16(5):581. doi: 10.3390/genes16050581 (PMC12111319; doi:10.3390/genes16050581)
Supplement: Supplementary file 1 [file genes-16-00581-s001.zip › genes-3620926-supplementary.pdf]

Genetic determinants of colonic diverticulosis (PRISMA)

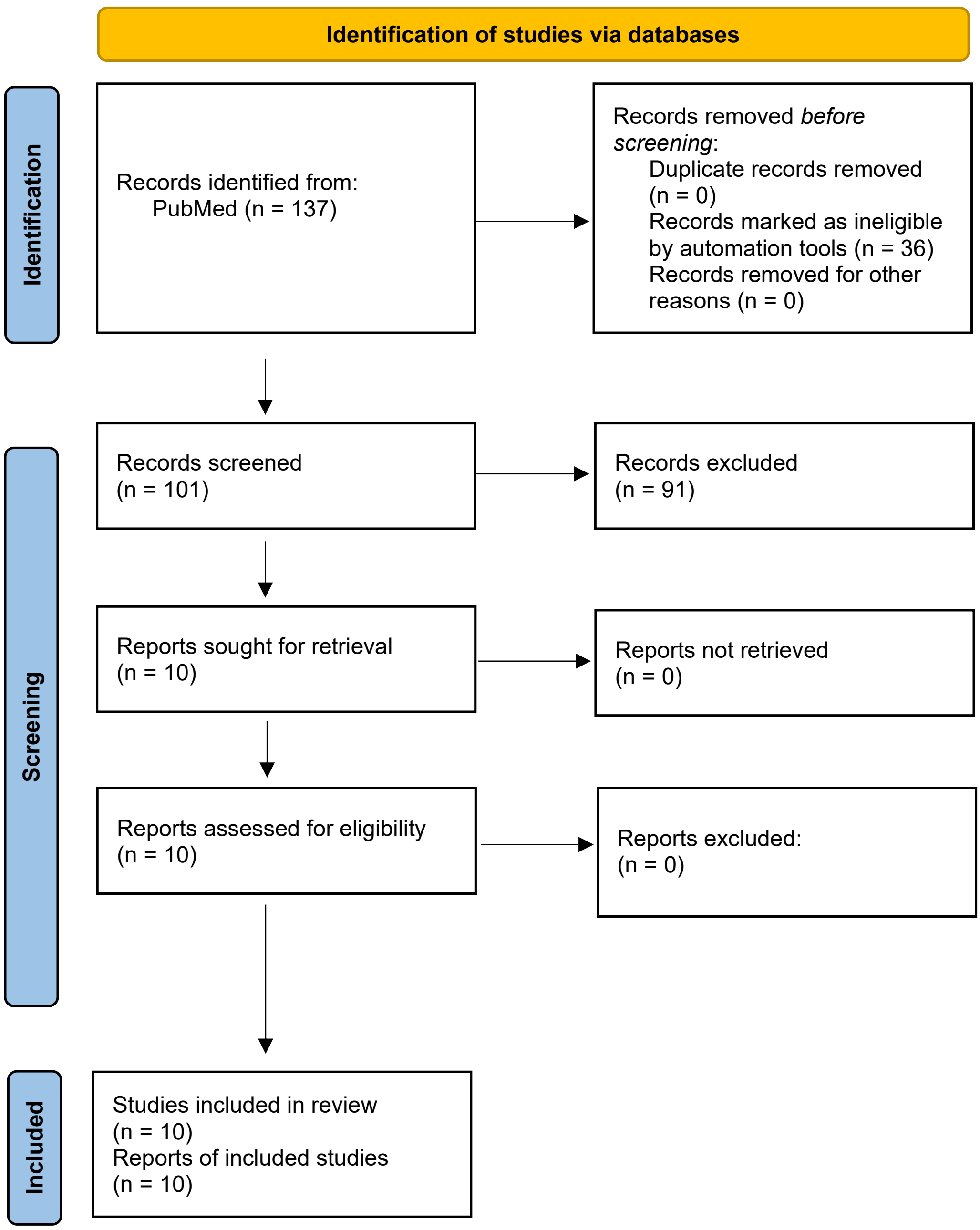

12. Page, M.J.; McKenzie, J.E.; Bossuyt, P.M.; Boutron, I.; Hoffmann, T.C.; Mulrow, C.D.; Shamseer, L.; Tetzlaff, J.M.; Akl, E.A.; Brennan, S.E.; et al. The PRISMA 2020 statement: an updated guideline for reporting systematic reviews. *BMJ* **2021**, *372*, n71. https://doi.org/10.1136/bmj.n71

For more information, visit: http://www.prisma-statement.org/
